# Health risk assessment for severe COVID-19 in Taiwan: a multi-centre electronic health record study

**DOI:** 10.7189/jogh.15.04236

**Published:** 2025-09-05

**Authors:** Yu-Hui Chang, Whitney Burton, Phung-Anh Nguyen, Do Duy Khang, Chang-I Chen, Chung-Chien Huang, Carlos Shu-Kei Lam, Wen-Kuang Lin, Fu-Der Wang, Phan Thanh Phuc, Christine Y Lu, Hsin-Lun Lee, Min-Huei Hsu, Chih-Wei Huang, Hsuan-Chia Yang, Shiue-Ming Lin, Chieh Yang, Jason C Hsu

**Affiliations:** 1 School of Pharmacy, College of Pharmacy, Taipei Medical University, Taipei, Taiwan; 2 International Ph.D. Program in Biotech and Healthcare Management, College of Management, Taipei Medical University, Taipei, Taiwan; 3 Graduate Institute of Data Science, College of Management, Taipei Medical University, Taipei City, Taiwan; 4 Clinical Data Center, Office of Data Science, Taipei Medical University, Taipei, Taiwan; 5 Clinical Big Data Research Center, Taipei Medical University Hospital, Taipei Medical University, Taipei, Taiwan; 6 Research Center of Data Science on Health Care Industry, College of Management, Taipei Medical University, Taipei, Taiwan; 7 Ph.D. Program in Global Health and Health Security, School of Public Health, Taipei Medical University, Taipei, Taiwan; 8 Department of Healthcare Administration, School of Management, Taipei Medical University, Taipei, Taiwan; 9 Emergency Department, Department of Emergency and Critical Care Medicine, Wan Fang Hospital, Taipei Medical University, Taipei, Taiwan; 10 Department of Emergency, School of Medicine, College of Medicine, Taipei Medical University, Taipei, Taiwan; 11 Graduate Institute of Injury Prevention and Control, College of Public Health, Taipei Medical University, Taipei, Taiwan; 12 Division of Infectious Diseases, Department of Internal Medicine, Taipei Medical University Hospital, Taipei, Taiwan; 13 Institute of Public Health, National Yang-Ming Chiao-Tung University, Taipei, Taiwan; 14 Kolling Institute, Faculty of Medicine and Health, The University of Sydney and the Northern Sydney Local Health District, Sydney, NSW, Australia; 15 School of Pharmacy, Faculty of Medicine and Health, The University of Sydney, Sydney, New South Wales, Australia; 16 Department of Radiology, School of Medicine, College of Medicine, Taipei Medical University, Taipei, Taiwan; 17 Department of Radiation Oncology, Taipei Medical University Hospital, Taipei, Taiwan; 18 Office of Data Science, Taipei Medical University, Taipei, Taiwan; 19 International Center for Health Information Technology, Taipei Medical University, Taipei, Taiwan; 20 Graduate Institute of Biomedical Informatics, College of Medical Science and Technology, Taipei Medical University, Taipei, Taiwan; 21 Research Center of Big Data and Meta-analysis, Wanfang Hospital, Taipei Medical University, Taipei, Taiwan

## Abstract

**Background:**

As the global battle against COVID-19 continues, understanding the factors contributing to severe outcomes remains critical for public health strategies. We aim to identify the determinants significantly influencing severe COVID-19 infection and mortality among the general population in Taiwan.

**Methods:**

We conducted a retrospective cohort study using data extracted from the Taipei Medical University Clinical Research Database from 1 January 2022 to 31 December 2022. We defined the primary outcomes as severe COVID-19 infection, including hospitalisation, ventilator use, intubation, and mortality. We performed logistic regression analyses to explore the association of various factors, including demographic characteristics, body mass index (BMI), Charlson Comorbidity Index score, and multiple comorbidities.

**Results:**

Among 96 489 confirmed COVID-19 cases, 44 996 (46.6%) were classified as high-risk patients. Compared to females, male patients had significantly higher risks of ventilator use (odds ratio (OR) = 1.245; 95% confidence interval (CI) = 1.147–1.352, *P* < 0.0001), intubation (OR = 1.115; 95% CI = 1.011–1.230, *P* = 0.03), and mortality (OR = 1.510; 95% CI = 1.332–1.713, *P* < 0.0001). Patients with lower BMI had significantly increased risks of ventilator use (OR = 0.972; 95% CI = 0.964–0.981, *P* < 0.0001) and mortality (OR = 0.92; 95% CI = 0.908–0.935, *P* < 0.0001), compared to patients with higher BMI. Patients with chronic comorbidities such as heart disease, moderate to severe kidney disease, diabetes, cancer, hypertension, anaemia, and Parkinson disease had significantly higher risks of severe COVID-19 or mortality compared to those without these conditions. Conversely, patients with peptic ulcer disease or hyperlipidaemia seem to have lower risks of severity and mortality compared to those without these conditions.

**Conclusions:**

We found that being male, having a lower BMI, and having certain chronic conditions increased the risk of severe COVID-19 outcomes, while peptic ulcer disease and hyperlipidaemia were linked to reduced risks. These findings highlight the need for targeted public health strategies for high-risk groups.

The COVID-19 pandemic has presented an unprecedented challenge to global health. Taiwan's response to the pandemic has been noted for its early success in management. However, the region faces ongoing challenges due to its high population density and the dynamic nature of the virus. In early 2022, a mutated variant of SARS-CoV-2, *Omicron*, emerged and quickly surged worldwide. The *Omicron* variant is the most heavily mutated, leading to increased transmissibility and partial resistance to immunity from COVID-19 vaccines [1]. Globally, it continues to affect millions, necessitating a deeper understanding of the factors that contribute to severe disease outcomes. Studies worldwide have investigated various factors of severe COVID-19 outcomes, focussing on demographic and clinical characteristics like age [2], gender [3], body mass index (BMI) [4], and existing health conditions [5–7].

Despite extensive research on the factors influencing severe COVID-19 outcomes in various countries, the impact of these factors can vary due to differences in ethnicity and region [8]. Research on the factors influencing severe COVID-19 outcomes in Taiwan is still limited. While there are various ways to measure severe COVID-19 outcomes, most studies have used a single indicator, usually mortality, rather than multiple indicators [6,9]. Additionally, past research has often had a limited scope, lacking a comprehensive evaluation of factors such as many specific comorbidities [10]. Furthermore, most studies have focussed on the overall population, with few specifically investigating high-risk or non-high-risk groups in depth.

Due to the limited number of studies that provide a comprehensive assessment of all potential risk factors and multiple severe outcomes, there remains a significant gap in understanding the high-risk patient population. Moreover, most existing research has been conducted using databases primarily from Western countries, leading to a lack of studies focussed on the Taiwanese population. To address this gap, we aim to identify and evaluate factors linked to severe COVID-19 outcomes in Taiwan, comparing high-risk and non-high-risk populations. We sought to confirm established risk factors and explore additional relevant variables. By focussing on high-risk groups, we aim to enhance the understanding of what increases the risk of severe outcomes, offering insights for public health strategies in Taiwan.

## METHODS

### Study design and data source

In this retrospective cohort study, we used data sourced from the Taipei Medical University (TMU) Clinical Research Database [11], gathering electronic medical records from 1998 to 2022, and including records of about 4.3 million patients from the TMU hospital, Shuang-Ho hospital, and Wan-Fang hospital in northern Taiwan.

### Cohort selection

**Figure 1:**
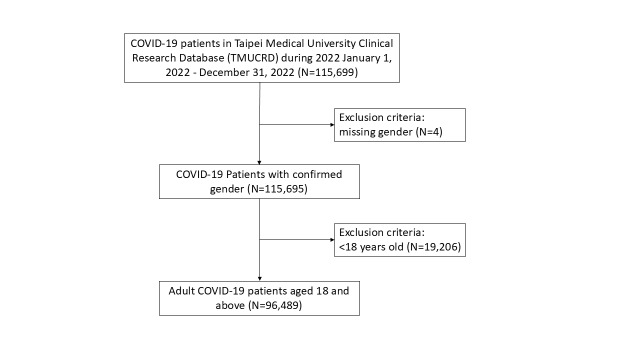
Cohort selection process.

We collected data on all patients with a positive SARS-CoV-2 PCR test from 1 January 2022 to 31 December 2022, excluding patients <18 years of age or with unspecified gender. We included 96 625 patients and retrieved their electronic medical records from 1 January 2011 onwards. (**Figure 1**)

### Outcome measurement

The index date was the first hospital admission due to COVID-19 infection between 1 January 2022 and 31 March 2022. Outcomes were based on severe outcomes of COVID-19, including hospitalisation, ventilator use, intubation, and mortality. We also conducted a sensitivity test by categorising patients into two groups according to the high-risk definition outlined in the Taiwan Centers for Disease Control guidelines [12]. We censored the data upon death, loss to follow-up, or at the end of the study period on 31 December 2022.

### Analysis of covariates

To select possible covariates, we conducted an extensive literature review and consulted with clinicians. These covariates included: demographic characteristics (*i.e.* gender and age), BMI, hospital-related variables (*i.e.* former patient at the TMU’s hospitals, vaccine at TMU’s hospitals, medical department, telemedicine), Charlson Comorbidity Index (CCI) score, comorbidities (*i.e.* myocardial infarction (MI), congestive heart failure (CHF), peripheral vascular disease (PVD), cerebrovascular disease (CVD), dementia, chronic pulmonary disease (COPD), rheumatic disease, peptic ulcer disease (PUD), hemiplegia or paraplegia, moderate/severe renal disease, liver disease, diabetes, cancer, hypertension, hyperlipidaemia, hyperuricemia, hyperthyroidism, depressive disorder and anxiety, anaemia, Parkinson disease and osteoporosis), and co-medications (*i.e.* nerve system, vascular system, metabolism).

We defined comorbidities using the International Classification of Diseases, 9th or 10th edition. We classified the patients as having comorbidities if their records showed at least two outpatient visits or one hospitalisation for the disease before the index date, and as long-term medication users if they were prescribed any of the previously mentioned drugs for at least 28 days in the year before the index date.

### Statistical analyses

We evaluated the baseline characteristics using one-way ANOVA for continuous variables and χ^2^ test for categorical variables. To establish associations between the covariates and outcomes, we used logistic regression, conducting both univariate and multivariate analyses. We performed a correlation analysis among all independent variables before conducting logistic regression to address collinearity concerns. To assess the potential for multicollinearity among variables, we calculated variance inflation factors (VIFs) for all independent variables (Tables S1 and S2 in the **Online Supplementary Document**). We reported the odds ratios (ORs), 95% confidence intervals (CIs), and *P*-values to indicate the risk associated with these variables. We conducted all analyses using SAS software, version 9.4 (SAS Institute Inc., Cary, North Carolina, USA).

## RESULTS

### Baseline characteristics

We included 115 699 patients diagnosed with COVID-19 at TMU’s hospitals in 2022 (**Figure 1**). After excluding four cases with missing gender and 19 206 patients <18 years, we analysed records from 96 489 patients. Of all, 44 996 (46.6%) patients were classified as high-risk.

Of 96 489 patients, 54.16% were female in the overall population and 53.89% in the high-risk group (**Table 1**). The mean (x̄) age of patients was higher in the high-risk group (x̄ = 54.91) compared to the overall population (x̄ = 46.69). Similarly, the BMI was higher in the high-risk group (x̄ = 25.45) compared to the overall population (x̄ = 24.14). While most cohort participants were generally healthy, the CCI score was slightly higher within the high-risk group. Among comorbidities, hypertension, hyperlipidaemia, and diabetes were the most prevalent. Additionally, patients in the high-risk group had a higher percentage of taking medication. The high-risk group also showed higher rates of all four severe COVID-19 outcomes.

**Table 1:** Basic characteristics of participants

	Overall	High risk
	**n (%)**	**n (%)**
**Total number of participants**	96 489	44 996
**Gender**		
Female	52 254 (54.16)	24 249 (53.89)
Male	44 235 (45.84)	20 747 (46.11)
**Age, x̄ (SD)**	46.69 (17.74)	54.91 (19.33)
**BMI, x̄ (SD)**	24.14 (4.92)	25.45 (5.85)
**Former patient at the three hospitals**		
No	11 652 (12.08)	2222 (4.94)
Yes	84 837 (87.92)	42 774 (95.06)
**Vaccine at the three hospitals**		
No	75 244 (77.98)	32 415 (72.04)
Yes	21 245 (22.02)	12 581 (27.96)
**Medical department**		
Others	35 447 (36.74)	14 170 (31.49)
Division of Emergency Medicine	54 898 (56.90)	28 054 (62.35)
Family Medicine	6144 (6.37)	2772 (6.16)
**Telemedicine**		
No	95 071 (98.53)	44 380 (98.63)
Yes	1418 (1.47)	616 (1.37)
**CCI score, x̄ (SD)**	0.49 (1.35)	1.00 (1.83)
**Comorbidities**		
MI	572 (0.59)	572 (1.27)
CHF	2309 (2.39)	2296 (5.10)
PVD	624 (0.65)	622 (1.38)
CVD	4265 (4.42)	4144 (9.21)
Dementia	1559 (1.62)	1559 (3.46)
COPD	4467 (4.63)	3922 (8.72)
Rheumatic disease	636 (0.66)	599 (1.33)
PUD	4691 (4.86)	3779 (8.40)
Hemiplegia or paraplegia	230 (0.24)	222 (0.49)
Moderate/severe renal disease	2840 (2.94)	2804 (6.23)
Liver disease	3188 (3.30)	2956 (6.57)
Diabetes	6420 (6.65)	6420 (14.27)
Cancer	2477 (2.57)	2393 (5.32)
Hypertension	10 939 (11.34)	10 393 (23.10)
Hyperlipidaemia	8463 (8.77)	7903 (17.56)
Hyperuricemia	1124 (1.16)	1017 (2.26)
Hyperthyroidism	782 (0.81)	559 (1.24)
Depressive disorder and anxiety	1659 (1.72)	1659 (3.69)
Anaemia	2576 (2.67)	2167 (4.82)
Parkinson disease	577 (0.60)	563 (1.25)
Osteoporosis	1173 (1.22)	1095 (2.43)
**Co-medications**		
Nerve	4930 (5.11)	4603 (10.23)
Vascular	8612 (8.93)	8372 (18.61)
Metabolism	9123 (9.45)	8800 (19.56)
Others	33 212 (34.42)	25 061 (55.70)
**Outcomes**		
Hospitalisation	4845 (5.02)	4229 (9.40)
Ventilator	3327 (3.45)	3121 (6.94)
Intubation	1786 (1.85)	1463 (3.25)
Mortality	1358 (1.41)	1317 (2.93)

BMI – body mass index, CCI – Charlson Comorbidity Index, CHF – congestive heart failure, COPD – chronic pulmonary disease, CVD – cerebrovascular disease, MI – myocardial infarction, PUD – peptic ulcer disease, PVD – peripheral vascular disease, SD – standard deviation, x̄ – mean

### Risk factors for severe COVID-19 outcomes among overall patients

#### Hospitalisation

**Figure 2:**
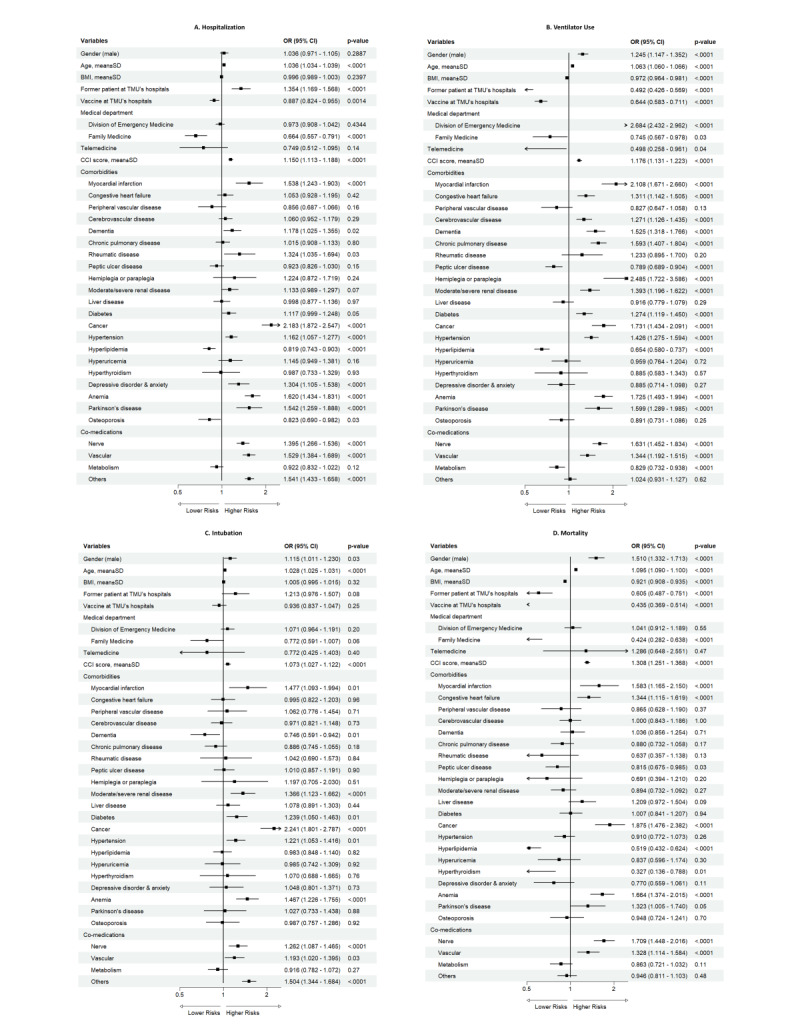
Risk factors of severe COVID-19 outcomes among overall patients. **Panel A.** Hospitalisation. **Panel B.** Ventilator use. **Panel C.** Intubation. **Panel D.** Mortality. Multivariable logistic regression models were used to estimate the ORs and 95% CIs for each outcome. All models were adjusted for age, sex, vaccination status, CCI score, and comorbidities. Variables with *P* < 0.05 were considered statistically significant. CI – confidence interval, ORs – odds ratios.

Older age (OR = 1.036; 95% CI = 1.034–1.039) was significantly associated with an increased risk of hospitalisation (**Figure 2**, **Table 2**). Regarding comorbidities, several conditions demonstrated a strong association with hospitalisation risk, including MI (OR = 1.538; 95% CI = 1.243–1.903), dementia (OR = 1.178; 95% CI = 1.025–1.355), rheumatic disease (OR = 1.324; 95% CI = 1.035–1.694), cancer (OR = 2.183; 95% CI = 1.872–2.547), hypertension (OR = 1.162; 95% CI = 1.057–1.277), depressive disorder and anxiety (OR = 1.304; 95% CI = 1.105–1.538), anaemia (OR = 1.620; 95% CI = 1.434–1.831), and Parkinson disease (OR = 1.542; 95% CI = 1.259–1.888). Additionally, using nerve (OR = 1.395; 95% CI = 1.266–1.536) and vascular (OR = 1.529; 95% CI = 1.384–1.689) co-medications seem to pose a higher risk for hospitalisation. Conversely, hyperlipidaemia (OR = 0.819; 95% CI = 0.743–0.903) and osteoporosis (OR = 0.823; 95% CI = 0.690–0.982) seem to decrease the risk for hospitalisation.

**Table 2:** Summary table of risk factors of outcomes*

	Overall population				High-risk population			
	**Hospitalisation**	**Ventilator**	**Intubation**	**Mortality**	**Hospitalisation**	**Ventilator**	**Intubation**	**Mortality**
**Gender (male)**	1.036 (0.971–1.105)	1.245 (1.147–1.352)	1.115 (1.011–1.230)	1.510 (1.332–1.713)	1.032 (0.962–1.108)	1.230 (1.129–1.340)	1.145 (1.026–1.277)	1.492 (1.313–1.695)
**Age, x̄ (SD)**	1.036 (1.034–1.039)	1.063 (1.060–1.066)	1.028 (1.025–1.031)	1.095 (1.090–1.100)	1.025 (1.023–1.028)	1.050 (1.047–1.053)	1.019 (1.015–1.023)	1.080 (1.074–1.085)
**BMI, x̄ (SD)**	0.996 (0.989–1.003)	0.972 (0.964–0.981)	1.005 (0.995–1.015)	0.921 (0.908–0.935)	0.980 (0.973–0.987)	0.963 (0.954–0.972)	0.990 (0.979–1.000)	0.915 (0.902–0.929)
**CCI score, x̄ (SD)**	1.150 (1.113–1.188)	1.176 (1.131–1.223)	1.073 (1.027–1.122)	1.308 (1.251–1.368)	1.148 (1.111–1.185)	1.167 (1.124–1.213)	1.080 (1.033–1.129)	1.287 (1.231–1.345)
**Comorbidities**								
MI	1.538 (1.243–1.903)	2.108 (1.671–2.660)	1.477 (1.093–1.994)	1.583 (1.165–2.150)	1.498 (1.216–1.846)	2.019 (1.610–2.533)	1.457 (1.081–1.963)	1.565 (1.159–2.114)
CHF	1.053 (0.928–1.195)	1.311 (1.142–1.505)	0.995 (0.822–1.203)	1.344 (1.115–1.619)	1.106 (0.977–1.252)	1.350 (1.180–1.545)	1.044 (0.864–1.262)	1.389 (1.156–1.669)
CVD	1.060 (0.952–1.179)	1.271 (1.126–1.435)	0.971 (0.821–1.148)	1.000 (0.843–1.186)	1.043 (0.938–1.158)	1.245 (1.106–1.401)	0.959 (0.812–1.133)	0.998 (0.844–1.180)
Dementia	1.178 (1.025–1.355)	1.525 (1.318–1.766)	0.746 (0.591–0.942)	1.036 (0.856–1.254)	1.271 (1.107–1.459)	1.621 (1.403–1.872)	0.806 (0.638–1.017)	1.118 (0.926–1.352)
COPD	1.015 (0.908–1.133)	1.593 (1.407–1.804)	0.886 (0.745–1.055)	0.880 (0.732–1.058)	1.002 (0.898–1.117)	1.555 (1.376–1.756)	0.875 (0.735–1.043)	0.904 (0.755–1.084)
PUD	0.923 (0.826–1.030)	0.789 (0.689–0.904)	1.010 (0.857–1.191)	0.815 (0.675–0.985)	0.911 (0.815–1.018)	0.788 (0.689–0.902)	0.962 (0.812–1.139)	0.828 (0.687–0.998)
Moderate/severe renal disease	1.133 (0.989–1.297)	1.393 (1.196–1.622)	1.366 (1.123–1.662)	0.894 (0.732–1.092)	1.153 (1.009–1.317)	1.405 (1.210–1.630)	1.367 (1.125–1.661)	0.931 (0.764–1.134)
Liver disease	0.998 (0.877–1.136)	0.916 (0.779–1.079)	1.078 (0.891–1.303)	1.209 (0.972–1.504)	0.936 (0.824–1.064)	0.861 (0.734–1.010)	1.024 (0.846–1.239)	1.139 (0.919–1.412)
Diabetes	1.117 (0.999–1.248)	1.274 (1.119–1.450)	1.239 (1.050–1.463)	1.007 (0.841–1.207)	1.090 (0.977–1.216)	1.224 (1.079–1.388)	1.222 (1.037–1.440)	0.997 (0.835–1.190)
Cancer	2.183 (1.872–2.547)	1.731 (1.434–2.091)	2.241 (1.801–2.787)	1.875 (1.476–2.382)	1.970 (1.690–2.297)	1.574 (1.307–1.895)	2.040 (1.636–2.544)	1.766 (1.394–2.238)
Hypertension	1.162 (1.057–1.277)	1.426 (1.275–1.594)	1.221 (1.053–1.416)	0.910 (0.772–1.073)	1.140 (1.038–1.251)	1.375 (1.233–1.534)	1.211 (1.045–1.404)	0.903 (0.768–1.061)
Hyperlipidaemia	0.819 (0.743–0.903)	0.654 (0.580–0.737)	0.983 (0.848–1.140)	0.519 (0.432–0.624)	0.788 (0.715–0.868)	0.647 (0.576–0.727)	0.938 (0.808–1.088)	0.521 (0.435–0.624)
Anaemia	1.620 (1.434–1.831)	1.725 (1.493–1.994)	1.467 (1.226–1.755)	1.664 (1.374–2.015)	1.552 (1.373–1.754)	1.691 (1.466–1.949)	1.450 (1.209–1.738)	1.667 (1.380–2.013)
Parkinson disease	1.542 (1.259–1.888)	1.599 (1.289–1.985)	1.027 (0.733–1.438)	1.323 (1.005–1.740)	1.596 (1.306–1.950)	1.626 (1.314–2.012)	1.083 (0.774–1.516)	1.358 (1.035–1.781)
**Co-medications**								
Nerve	1.395 (1.266–1.536)	1.631 (1.452–1.834)	1.262 (1.087–1.465)	1.709 (1.448–2.016)	1.355 (1.232–1.491)	1.566 (1.396–1.756)	1.218 (1.049–1.416)	1.660 (1.411–1.953)
Vascular	1.529 (1.384–1.689)	1.344 (1.192–1.515)	1.193 (1.020–1.395)	1.328 (1.114–1.584)	1.527 (1.384–1.685)	1.334 (1.187–1.500)	1.185 (1.014–1.385)	1.332 (1.120–1.583)
Metabolism	0.922 (0.832–1.022)	0.829 (0.732–0.938)	0.916 (0.782–1.072)	0.863 (0.721–1.032)	0.910 (0.823–1.008)	0.809 (0.717–0.912)	0.899 (0.768–1.052)	0.853 (0.715–1.016)

BMI – body mass index, CCI – Charlson Comorbidity Index, CHF – congestive heart failure, COPD – chronic pulmonary disease, CVD – cerebrovascular disease, MI – myocardial infarction, PUD – peptic ulcer disease, SD – standard deviation, x̄ – mean

*Values are presented as odds ratio (95% confidence interval).

#### Ventilator use

Male (OR = 1.245; 95% CI = 1.147–1.352), older age (OR = 1.063; 95% CI = 1.060–1.066), and higher CCI score (OR = 1.176; 95% CI = 1.131–1.223) were significantly associated with an increased risk of ventilator use (**Figure 2**, **Table 2**). Regarding comorbidities, several conditions demonstrated a strong association with ventilator use risk, including MI (OR = 2.108; 95% CI = 1.671–2.660), CHF (OR = 1.311; 95% CI = 1.142–1.505), CVD (OR = 1.271; 95% CI = 1.126–1.435), dementia (OR = 1.525; 95% CI = 1.318–1.766), COPD (OR = 1.593; 95% CI = 1.407–1.804), hemiplegia or paraplegia (OR = 2.485; 95% CI = 1.722–3.586), moderate/severe renal disease (OR = 1.393; 95% CI = 1.196–1.622), diabetes (OR = 1.274; 95% CI = 1.119–1.450), cancer (OR = 1.731; 95% CI = 1.434–2.091), hypertension (OR = 1.426; 95% CI = 1.275–1.594), anaemia (OR = 1.725; 95% CI = 1.493–1.994), and Parkinson disease (OR = 1.599; 95% CI = 1.289–1.985). Additionally, using nerve (OR = 1.631; 95% CI = 1.452–1.834) and vascular (OR = 1.344; 95% CI = 1.192–1.515) co-medications were associated with a higher risk of ventilator use. Conversely, higher BMI (OR = 0.972; 95% CI = 0.964–0.981), PUD (OR = 0.789; 95% CI = 0.689–0.904), hyperlipidaemia (OR = 0.654; 95% CI = 0.580–0.737), and metabolism co-medications (OR = 0.829; 95% CI = 0.732–0.938) were associated with decreased risk of ventilator use.

#### Intubation

Male (OR = 1.115; 95% CI = 1.011–1.230), older age (OR = 1.028; 95% CI = 1.025 − 1.031), and higher CCI score (OR = 1.073; 95% CI = 1.027–1.122) were significantly associated with an increased risk of intubation (**Figure 2**, **Table 2**). Regarding comorbidities, several conditions demonstrated a strong association with intubation risk, including MI (OR = 1.477; 95% CI = 1.093–1.994), moderate/severe renal disease (OR = 1.366; 95% CI = 1.123–1.662), diabetes (OR = 1.239; 95% CI = 1.050–1.463), cancer (OR = 2.241; 95% CI = 1.801–2.787), hypertension (OR = 1.221; 95% CI = 1.053–1.416), and anaemia (OR = 1.467; 95% CI = 1.226–1.755). Additionally, using nerve (OR = 1.262; 95% CI = 1.087–1.465) and vascular (OR = 1.193; 95% CI = 1.020–1.395) co-medications were associated with a higher risk of intubation. Conversely, dementia (OR = 0.746; 95% CI = 0.591–0.942) was associated with a decreased risk of intubation.

#### Mortality

Male (OR = 1.510; 95% CI = 1.332–1.713), older age (OR = 1.095; 95% CI = 1.090–1.100), and higher CCI score (OR = 1.308; 95% CI = 1.251–1.368) were significantly associated with an increased risk of mortality (**Figure 2**, **Table 2**). Regarding comorbidities, several conditions demonstrated a strong association with intubation risk, including MI (OR = 1.583; 95% CI = 1.165–2.150), CHF (OR = 1.344; 95% CI = 1.115–1.619), cancer (OR = 1.875; 95% CI = 1.476–2.382), and anaemia (OR = 1.664; 95% CI = 1.374–2.015). Additionally, using nerve (OR = 1.709; 95% CI = 1.448–2.016) and vascular (OR = 1.328; 95% CI = 1.114–1.584) co-medications were associated with a higher risk of mortality. Conversely, higher BMI (OR = 0.921; 95% CI = 0.908–0.935), PUD (OR = 0.815; 95% CI = 0.675–0.985), hyperlipidaemia (OR = 0.519; 95% CI = 0.432–0.624), and hyperthyroidism (OR = 0.327; 95% CI = 0.136–0.788) were associated with a decreased mortality risk.

### Subgroup analysis of high-risk patients

#### Hospitalisation

**Figure 3:**
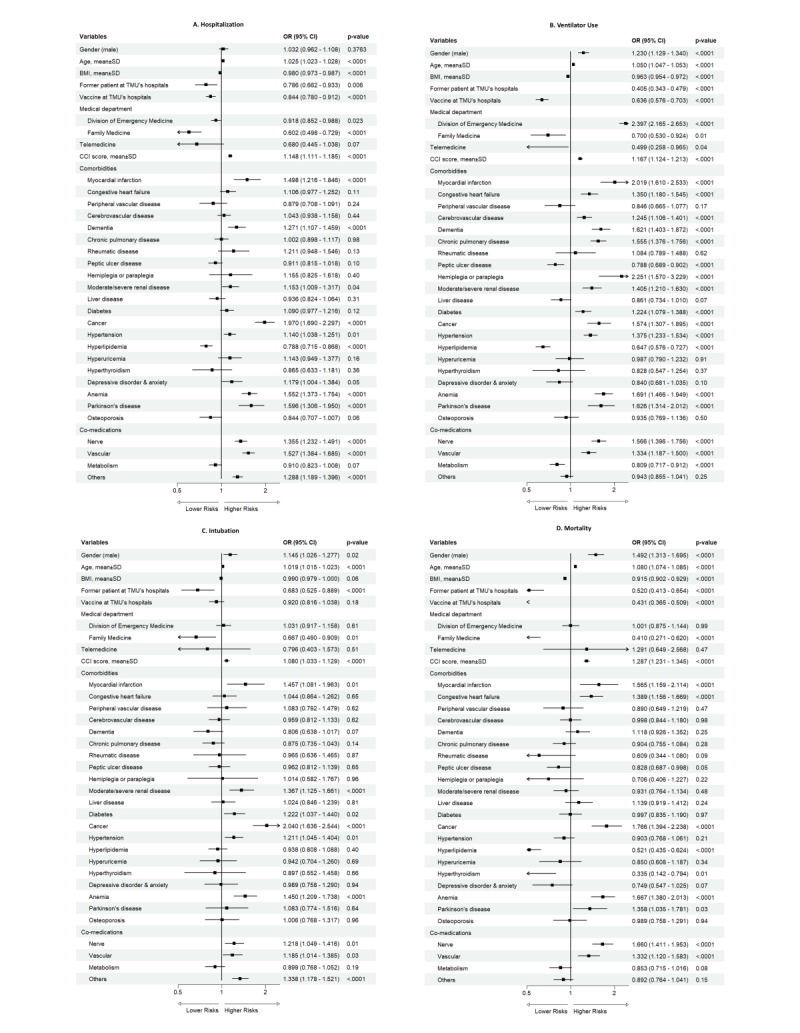
Risk factors of severe COVID-19 outcomes among high-risk patients. **Panel A.** Hospitalisation. **Panel B.** Ventilator use. **Panel C.** Intubation. **Panel D.** Mortality. Multivariable logistic regression models were used to estimate the ORs and 95% CIs for each outcome. All models were adjusted for age, sex, vaccination status, CCI score, and comorbidities. Variables with *P* < 0.05 were considered statistically significant. CI – confidence interval, ORs – odds ratios.

Older age (OR = 1.025; 95% CI = 1.023–1.028) and higher CCI score (OR = 1.148; 95% CI = 1.111–1.185) were significantly associated with an increased risk of hospitalisation (**Figure 3**, **Table 2**). Regarding comorbidities, several conditions demonstrated a strong association with hospitalisation risk, including MI (OR = 1.498; 95% CI = 1.216–1.846), dementia (OR = 1.271; 95% CI = 1.107–1.459), moderate/severe renal disease (OR = 1.153; 95% CI = 1.009–1.317), cancer (OR = 1.970; 95% CI = 1.690–2.297), hypertension (OR = 1.140; 95% CI = 1.038–1.251), anaemia (OR = 1.552; 95% CI = 1.373–1.754), and Parkinson disease (OR = 1.596; 95% CI = 1.306–1.950). Additionally, using nerve (OR = 1.355; 95% CI = 1.232–1.491) and vascular (OR = 1.527; 95% CI = 1.384–1.685) co-medications were associated with a higher risk of hospitalisation. Conversely, higher BMI (OR = 0.980; 95% CI = 0.973–0.987) and hyperlipidaemia (OR = 0.788; 95% CI = 0.715–0.868) were associated with a decreased risk of hospitalisation.

#### Ventilator use

Male (OR = 1.230; 95% CI = 1.129–1.340), older age (OR = 1.050; 95% CI = 1.047–1.053), and higher CCI score (OR = 1.167; 95% CI = 1.124–1.213) were significantly associated with an increased risk of ventilator use (**Figure 3**, **Table 2**). Regarding comorbidities, several conditions demonstrated a strong association with ventilator use risk, including MI (OR = 2.019; 95% CI = 1.610–2.533), CHF (OR = 1.350; 95% CI = 1.180–1.545), CVD (OR = 1.245; 95% CI = 1.106–1.401), dementia (OR = 1.621; 95% CI = 1.403–1.872), COPD (OR = 1.555; 95% CI = 1.376–1.756), hemiplegia or paraplegia (OR = 2.251; 95% CI = 1.570–3.229), moderate/severe renal disease (OR = 1.405; 95% CI = 1.210–1.630), diabetes (OR = 1.224; 95% CI = 1.079–1.388), cancer (OR = 1.574; 95% CI = 1.307–1.895), hypertension (OR = 1.375; 95% CI = 1.233–1.534), anaemia (OR = 1.691; 95% CI = 1.466–1.949), and Parkinson disease (OR = 1.626; 95% CI = 1.314–2.012). Additionally, using nerve (OR = 1.566; 95% CI = 1.396–1.756) and vascular (OR = 1.334; 95% CI = 1.187–1.500) co-medications were associated with a higher risk of ventilator use. Conversely, higher BMI (OR = 0.963; 95% CI = 0.954–0.972), PUD (OR = 0.788; 95% CI = 0.689–0.902), hyperlipidaemia (OR = 0.647; 95% CI = 0.576–0.727), and metabolism co-medications (OR = 0.809; 95% CI = 0.717–0.912) were associated with a decreased risk of ventilator use.

#### Intubation

Male (OR = 1.145; 95% CI = 1.026–1.277), older age (OR = 1.019; 95% CI = 1.015–1.023), and higher CCI score (OR = 1.080; 95% CI = 1.033–1.129) were significantly associated with an increased risk of intubation (**Figure 3**, **Table 2**). Regarding comorbidities, several conditions demonstrated a strong association with intubation risk, including MI (OR = 1.457; 95% CI = 1.081–1.963), moderate/severe renal disease (OR = 1.367; 95% CI = 1.125–1.661), diabetes (OR = 1.222; 95% CI = 1.037–1.440), cancer (OR = 2.040; 95% CI = 1.636–2.544), hypertension (OR = 1.211; 95% CI = 1.045–1.404), and anaemia (OR = 1.450; 95% CI = 1.209-1.738). Additionally, using nerve (OR = 1.218; 95% CI = 1.049–1.416) and vascular (OR = 1.185; 95% CI = 1.014–1.385) co-medications were associated with a higher risk of intubation.

#### Mortality

Male (OR = 1.492; 95% CI = 1.313–1.695), older age (OR = 1.080; 95% CI = 1.074–1.085), and higher CCI score (OR = 1.287; 95% CI = 1.231–1.345) were significantly associated with an increased risk of mortality (**Figure 3**, **Table 2**). Regarding comorbidities, several conditions demonstrated a strong association with mortality risk, including MI (OR = 1.565; 95% CI = 1.159–2.114), CHF (OR = 1.389; 95% CI = 1.156–1.669), cancer (OR = 1.766; 95% CI = 1.394–2.238), anaemia (OR = 1.667; 95% CI = 1.380–2.013), and Parkinson disease (OR = 1.358; 95% CI = 1.035–1.781). Additionally, using nerve (OR = 1.660; 95% CI = 1.411–1.953) and vascular (OR = 1.332; 95% CI = 1.120–1.583) co-medications were associated with a higher mortality risk. Conversely, higher BMI (OR = 0.915; 95% CI = 0.902–0.929), hyperlipidaemia (OR = 0.521; 95% CI = 0.435–0.624), and hyperthyroidism (OR = 0.335; 95% CI = 0.142–0.794) were associated with a decreased mortality risk.

## DISCUSSION

This study represents the first observational analysis in Taiwan using real-world data from multiple centres to comprehensively investigate factors contributing to severe COVID-19 infection and mortality among overall and high-risk patients. Our findings demonstrate that several factors were associated with a poor prognosis, including being male, older age, having lower BMI, higher CCI scores, and certain chronic conditions. Additionally, the use of certain medications, particularly for nerve and vascular conditions, was linked to an increased risk of adverse outcomes. Conversely, PUD and hyperlipidaemia were identified as beneficial factors in reducing risks.

Consistent with prior research, our study revealed that men displayed a higher risk of severe infection and mortality compared to women [13]. Previous research shows females exhibit a more rapid and robust immune response than males, characterised by higher CD4 + T cell counts and increased production of immunoglobulins within B cells [14]. Additionally, we observed a correlation between patients' age and prognosis. As age advances, prior studies have noted both a rise in poor outcomes due to the weakening immune system and increased COVID-19 vulnerability among older adults due to the age-related decline in immune defence [15,16].

Two studies [17,18] found that COVID-19 patients with BMIs ≥20 faced higher risks of hospitalisation and mortality, even after vaccination, whereas our results suggest that higher BMI may instead be associated with a protective effect. The potential explanation for this inconsistency may be the high prevalence of missing values for height or weight in the medical records. In addition, the previous study in the USA indicated a positive correlation between obesity rates and COVID-19 mortality, especially in populations with low vaccination coverage, minimal mask mandates, relaxed gathering restrictions, or lower household incomes [19]. However, Taiwan had high vaccination coverage, prioritised vaccination for high-risk groups, including the obese, enforced strict mask mandates, and maintained equitable healthcare access through national insurance.

In line with the results of a previous meta-analysis [20], we found that a higher CCI score increased the risk of mortality and severe outcomes. Patients with higher CCI scores tend to have more chronic diseases, increasing the risk of poor prognosis. Similar to a study that proved heart failure was associated with a higher risk of ventilator usage and mortality [21], our study extends beyond hospitalised patients, resulting in more robust findings. Another study showed that chronic kidney diseases significantly increase mortality, while we found that it only increased the risk of ventilator and intubation. Additionally, hypertension and diabetes have been observed to be prevalent among hospitalised COVID-19 patients based on early data [22]. Our findings also demonstrated that hypertension and diabetes are risk factors for severe outcomes, although not significantly associated with mortality. Similar to a meta-analysis that found anaemia associated with a significantly elevated risk for mortality [23], our findings also showed an increased risk of hospitalisation, intubation, and ventilator use. Cancer tends to be associated with severe outcomes in our study. Consistently, previous research indicates that malignancy correlates with a higher risk of critical COVID-19 outcomes [24]. Similar to the findings from a meta-analysis [25], we also found Parkinson disease to increase the risk of hospitalisation and mortality, as well as ventilation usage.

The findings from previous studies consistently indicate that metabolic abnormalities are associated with an increased risk of poor progression of COVID-19 [26,27]. Our study showed contradictory results, indicating a lower risk of hospitalisation, ventilator use, and mortality. However, another study highlights the importance of managing the health conditions of individuals with metabolic abnormalities to reduce the occurrence of severe COVID-19 by vaccination [27]. Thus, the potential explanation for this inconsistency lies in considering potential confounding variables, such as medication usage and vaccination status. A meta-analysis found that PUD is associated with a higher risk of COVID-19 severity in older patients but not in younger ones [28]. In contrast, our results showed that PUD reduced the risk of ventilator usage and mortality across both the overall population and the high-risk group. This discrepancy may be attributed to differences in sample sizes or the routine use of acid-suppressive therapy, such as H2 blockers or proton pump inhibitors, among PUD patients. A study has reported that medications like famotidine and pantoprazole are linked to reduced all-cause mortality, with famotidine additionally associated with lower odds of mechanical ventilation, vasopressor use, acute kidney injury, and gastrointestinal bleeding in hospitalised COVID-19 patients [29]. However, other studies have indicated that proton pump inhibitor use may increase the risk of severe COVID-19 outcomes [30]. These conflicting findings highlight the need for further research into the role of acid-suppressive therapy in COVID-19 prognosis.

### Limitations and future research

By using a comprehensive data set from multiple hospitals, conducting rigorous statistical analyses, and incorporating a wide range of variables, we enhanced the generalisability and robustness of our study. Additionally, due to the high transmissibility of the *Omicron* variant, our research is crucial and clinically significant. However, several limitations should be noted, including potential data incompleteness, misclassification of diagnoses, missing vaccination records, the constrained study period, and the absence of social determinants variables. Since the study period primarily coincided with the *Omicron* variant outbreak, further research is needed to assess the findings across different variants. Additionally, although we identified many risk factors for clinical use, the underlying rationale requires further validation through prospective studies to supplement and confirm our findings. Furthermore, we derived our study population from three hospitals in northern Taiwan; the generalisability to other countries or regions with more diverse populations may be limited.

## CONCLUSIONS

Being male, having a lower BMI, and having certain chronic conditions increased the risk of severe COVID-19 outcomes, while PUD and hyperlipidaemia reduced risks. These findings provide valuable insights into clinical decision-making and public health interventions in Taiwan. Optimising treatment strategies reduces severe illness and mortality, thereby alleviating the burden on the healthcare system. However, since we sourced data from the *Omicron* period, more research may be needed to improve generalisability for other viral variants.

## Additional material


Online Supplementary Document
