# Supplementary material for: Health risk assessment for severe COVID-19 in Taiwan: a multi-centre electronic health record study
Source: J Glob Health. 2025 Sep 5;15:04236. doi: 10.7189/jogh.15.04236 (PMC12412269; doi:10.7189/jogh.15.04236)
Supplement: Online Supplementary Document [file jogh-15-04236-s001.pdf]

Supplement to: Chang Y, Burton W, Nguyen P, Khang D, Chen C, Huang C, Lam CS, Lin W, Wang F, Phuc PT, Lu CY, Lee H, Hsu M, Huang C, Yang H, Lin S, Yang C, Hsu JC. Health risk assessment for severe COVID-19 in Taiwan: a multi-centre electronic health record study. J Glob Health. 2025;15: 04236.

Table S1. Colinearity tests of tolerance and VIF of Outcomes among Overall Patients

	Hospitalization					Ventilator					Intubation					Mortality				
**Variables**	**DF**	**Parameter Estimate**	**Standard Error**	**Tolerance**	**VIF**	**DF**	**Parameter Estimate**	**Standard Error**	**Tolerance**	**VIF**	**DF**	**Parameter Estimate**	**Standard Error**	**Tolerance**	**VIF**	**DF**	**Parameter Estimate**	**Standard Error**	**Tolerance**	**VIF**
**Age**	1	0.00126	0.00004248	0.75446	1.32546	1	0.00141	3.448E-05	0.75446	1.32546	1	0.00040266	2.767E-05	0.75446	1.32546	1	0.0008236	0.00002309	0.75446	1.32546
**BMI**	1	-0.00046052	0.00013553	0.96588	1.03533	1	-0.00108	0.00011	0.96588	1.03533	1	-1.604E-05	8.828E-05	0.96588	1.03533	1	-0.00101	0.00007366	0.96588	1.03533
**CCI score**	1	0.03002	0.00145	0.11249	8.88955	1	0.02034	0.00118	0.11249	8.88955	1	0.00857	0.0009446	0.11249	8.88955	1	0.02492	0.00078822	0.11249	8.88955
**Gender (male)**	1	0.00297	0.00134	0.96166	1.03987	1	0.00739	0.00109	0.96166	1.03987	1	0.00228	0.0008728	0.96166	1.03987	1	0.00646	0.0007283	0.96166	1.03987
**Former patient at TMU's hospitals**	1	0.00139	0.00214	0.88387	1.13139	1	-0.01839	0.00173	0.88387	1.13139	1	-3.495E-05	0.00139	0.88387	1.13139	1	-0.00591	0.00116	0.88387	1.13139
**Vaccine at TMU's hospitals**	1	-0.00658	0.00163	0.94354	1.05984	1	-0.01255	0.00132	0.94354	1.05984	1	-0.0016	0.00106	0.94354	1.05984	1	-0.00974	0.00088414	0.94354	1.05984
**Medical department (Other as ref.)**																				
Division of Emergency Medicine	1	-0.00284	0.00145	0.82613	1.21047	1	0.02499	0.00118	0.82613	1.21047	1	0.00059755	0.0009475	0.82613	1.21047	1	0.00158	0.00079061	0.82613	1.21047
Family Medicine	1	-0.01464	0.0031	0.74771	1.33741	1	-0.004	0.00252	0.74771	1.33741	1	-0.00396	0.00202	0.74771	1.33741	1	-0.0061	0.00169	0.74771	1.33741
**Telemedicine**	1	-0.00906	0.00605	0.8078	1.23793	1	-0.00896	0.00491	0.8078	1.23793	1	-0.00318	0.00394	0.8078	1.23793	1	0.00116	0.00329	0.8078	1.23793
**Comorbidities**																				
Myocardial infarction	1	0.06203	0.00894	0.90962	1.09936	1	0.10667	0.00726	0.90962	1.09936	1	0.02753	0.00582	0.90962	1.09936	1	0.0312	0.00486	0.90962	1.09936
Congestive heart failure	1	0.01773	0.00495	0.7476	1.33761	1	0.05899	0.00402	0.7476	1.33761	1	0.00285	0.00323	0.7476	1.33761	1	0.02318	0.00269	0.7476	1.33761
Peripheral vascular disease	1	-0.01954	0.00851	0.92154	1.08514	1	-0.01324	0.00691	0.92154	1.08514	1	0.00385	0.00554	0.92154	1.08514	1	-0.00847	0.00462	0.92154	1.08514
Cerebrovascular disease	1	-0.00144	0.00385	0.68489	1.46008	1	0.024	0.00312	0.68489	1.46008	1	-0.00494	0.00251	0.68489	1.46008	1	-0.0107	0.00209	0.68489	1.46008
Dementia	1	0.06479	0.00585	0.78906	1.26733	1	0.14545	0.00474	0.78906	1.26733	1	-0.00997	0.00381	0.78906	1.26733	1	0.05542	0.00318	0.78906	1.26733
Chronic pulmonary disease	1	-0.0117	0.00355	0.77056	1.29776	1	0.0282	0.00288	0.77056	1.29776	1	-0.00868	0.00231	0.77056	1.29776	1	-0.01628	0.00193	0.77056	1.29776
Rheumatic disease	1	0.00071273	0.00828	0.95528	1.04681	1	-0.01038	0.00672	0.95528	1.04681	1	-0.00783	0.00539	0.95528	1.04681	1	-0.03909	0.0045	0.95528	1.04681
Peptic ulcer disease	1	-0.02183	0.00352	0.74936	1.33448	1	-0.02282	0.00285	0.74936	1.33448	1	-0.00439	0.00229	0.74936	1.33448	1	-0.02418	0.00191	0.74936	1.33448
Hemiplegia or paraplegia	1	0.01168	0.01397	0.92427	1.08194	1	0.09687	0.01133	0.92427	1.08194	1	-0.00172	0.0091	0.92427	1.08194	1	-0.05005	0.00759	0.92427	1.08194
Moderate/severe renal disease	1	0.01645	0.00552	0.49196	2.0327	1	0.05945	0.00448	0.49196	2.0327	1	0.01783	0.0036	0.49196	2.0327	1	-0.01229	0.003	0.49196	2.0327
Liver disease	1	-0.02421	0.0041	0.79766	1.25367	1	-0.02564	0.00333	0.79766	1.25367	1	-0.00523	0.00267	0.79766	1.25367	1	-0.01844	0.00223	0.79766	1.25367
Diabetes	1	-0.00618	0.00397	0.43853	2.28033	1	0.01251	0.00322	0.43853	2.28033	1	0.00328	0.00258	0.43853	2.28033	1	-0.0213	0.00216	0.43853	2.28033
Cancer	1	0.08456	0.00637	0.422	2.36969	1	0.03729	0.00517	0.422	2.36969	1	0.03959	0.00415	0.422	2.36969	1	0.0148	0.00346	0.422	2.36969
Hypertension	1	0.01376	0.0029	0.50831	1.96731	1	0.02497	0.00235	0.50831	1.96731	1	0.00691	0.00189	0.50831	1.96731	1	-0.0003828	0.00157	0.50831	1.96731
Hyperlipidemia	1	-0.02631	0.00303	0.58498	1.70946	1	-0.03826	0.00246	0.58498	1.70946	1	-0.00346	0.00197	0.58498	1.70946	1	-0.02419	0.00164	0.58498	1.70946
Hyperuricemia	1	0.01457	0.00631	0.93376	1.07094	1	0.00086546	0.00512	0.93376	1.07094	1	-6.605E-05	0.00411	0.93376	1.07094	1	-0.00595	0.00343	0.93376	1.07094
Hyperthyroidism	1	-0.00914	0.00733	0.99151	1.00856	1	-0.01141	0.00595	0.99151	1.00856	1	-0.00102	0.00478	0.99151	1.00856	1	-0.01315	0.00399	0.99151	1.00856
Depressive disorder & anxiety	1	0.01689	0.00516	0.95277	1.04957	1	-0.0132	0.00419	0.95277	1.04957	1	0.00056853	0.00336	0.95277	1.04957	1	-0.01067	0.0028	0.95277	1.04957
Anemia	1	0.05797	0.00428	0.90136	1.10944	1	0.04782	0.00347	0.90136	1.10944	1	0.02147	0.00279	0.90136	1.10944	1	0.03022	0.00233	0.90136	1.10944
Parkinson's disease	1	0.10122	0.00889	0.91249	1.0959	1	0.10694	0.00721	0.91249	1.0959	1	0.00839	0.00579	0.91249	1.0959	1	0.04855	0.00483	0.91249	1.0959
Osteoporosis	1	-0.00402	0.00614	0.94763	1.05526	1	0.00778	0.00498	0.94763	1.05526	1	0.00365	0.004	0.94763	1.05526	1	0.00466	0.00334	0.94763	1.05526
**Co-medications**																				
Nerve	1	0.03476	0.00329	0.81524	1.22664	1	0.02858	0.00267	0.81524	1.22664	1	0.01033	0.00215	0.81524	1.22664	1	0.01759	0.00179	0.81524	1.22664

Vascular	1	0.04989	0.00327	0.49333	2.02703	1	0.01953	0.00265	0.49333	2.02703	1	0.00925	0.00213	0.49333	2.02703	1	0.01052	0.00178	0.49333	2.02703
Metabolism	1	-0.01615	0.00333	0.45218	2.2115	1	-0.02598	0.0027	0.45218	2.2115	1	-0.0068	0.00217	0.45218	2.2115	1	-0.01429	0.00181	0.45218	2.2115
Others	1	0.01639	0.00157	0.77411	1.2918	1	-0.00601	0.00127	0.77411	1.2918	1	0.00628	0.00102	0.77411	1.2918	1	-0.00206	0.00085132	0.77411	1.2918

Table S2. Colinearity tests of tolerance and VIF of Outcomes among High Risk Patients

	Hospitalization					Ventilator					Intubation					Mortality				
Variables	DF	Parameter Estimate	Standard Error	Tolerance	VIF	DF	Parameter Estimate	Standard Error	Tolerance	VIF	DF	Parameter Estimate	Standard Error	Tolerance	VIF	DF	Parameter Estimate	Standard Error	Tolerance	VIF
**Age**	1	0.00153	0.00007866	0.71976	1.38935	1	0.00187	0.0000662	0.71976	1.38935	1	0.00045675	0.0000502	0.71976	1.38935	1	0.00111	0.0000455	0.71976	1.38935
**BMI**	1	-0.00103	0.00023166	0.90537	1.10452	1	-0.00145	0.0001949	0.90537	1.10452	1	-0.0002108	0.0001478	0.90537	1.10452	1	-0.00143	0.00013399	0.90537	1.10452
**CCI score**	1	0.02936	0.00197	0.12913	7.74412	1	0.02019	0.00165	0.12913	7.74412	1	0.00894	0.00125	0.12913	7.74412	1	0.02436	0.00114	0.12913	7.74412
**Gender (male)**	1	0.00437	0.00266	0.94468	1.05856	1	0.01296	0.00224	0.94468	1.05856	1	0.00464	0.0017	0.94468	1.05856	1	0.01219	0.00154	0.94468	1.05856
**Former patient at TMU's hospitals**	1	-0.02065	0.00629	0.89656	1.11538	1	-0.06773	0.00529	0.89656	1.11538	1	-0.01195	0.00401	0.89656	1.11538	1	-0.02966	0.00364	0.89656	1.11538
**Vaccine at TMU's hospitals**	1	-0.01355	0.00293	0.96283	1.03861	1	-0.02173	0.00246	0.96283	1.03861	1	-0.00265	0.00187	0.96283	1.03861	1	-0.01741	0.00169	0.96283	1.03861
**Medical department (Other as ref.)**																				
Division of Emergency Medicine	1	-0.00919	0.0029	0.8434	1.18568	1	0.04314	0.00244	0.8434	1.18568	1	0.00005154	0.00185	0.8434	1.18568	1	0.00034825	0.00168	0.8434	1.18568
Family Medicine	1	-0.03497	0.00622	0.74342	1.34514	1	-0.01047	0.00524	0.74342	1.34514	1	-0.01054	0.00397	0.74342	1.34514	1	-0.01546	0.0036	0.74342	1.34514
**Telemedicine**	1	-0.02206	0.01227	0.81792	1.22261	1	-0.02093	0.01033	0.81792	1.22261	1	-0.00504	0.00783	0.81792	1.22261	1	0.00175	0.0071	0.81792	1.22261
**Comorbidities**																				
Myocardial infarction	1	0.06021	0.01205	0.91309	1.09518	1	0.10237	0.01014	0.91309	1.09518	1	0.02646	0.00769	0.91309	1.09518	1	0.02898	0.00697	0.91309	1.09518
Congestive heart failure	1	0.01636	0.00671	0.76397	1.30896	1	0.05417	0.00564	0.76397	1.30896	1	0.00252	0.00428	0.76397	1.30896	1	0.02184	0.00388	0.76397	1.30896
Peripheral vascular disease	1	-0.02088	0.01147	0.92707	1.07867	1	-0.0156	0.00966	0.92707	1.07867	1	0.00347	0.00732	0.92707	1.07867	1	-0.00927	0.00664	0.92707	1.07867
Cerebrovascular disease	1	-0.0046	0.00529	0.71238	1.40375	1	0.02125	0.00445	0.71238	1.40375	1	-0.0063	0.00337	0.71238	1.40375	1	-0.01207	0.00306	0.71238	1.40375
Dementia	1	0.0586	0.00792	0.79264	1.2616	1	0.13379	0.00667	0.79264	1.2616	1	-0.01151	0.00506	0.79264	1.2616	1	0.04947	0.00458	0.79264	1.2616
Chronic pulmonary disease	1	-0.01211	0.0051	0.80368	1.24428	1	0.03327	0.00429	0.80368	1.24428	1	-0.01022	0.00326	0.80368	1.24428	1	-0.01533	0.00295	0.80368	1.24428
Rheumatic disease	1	-0.00295	0.01149	0.95904	1.04271	1	-0.01444	0.00967	0.95904	1.04271	1	-0.01	0.00733	0.95904	1.04271	1	-0.03945	0.00665	0.95904	1.04271
Peptic ulcer disease	1	-0.02361	0.00526	0.78315	1.2769	1	-0.02594	0.00442	0.78315	1.2769	1	-0.00671	0.00335	0.78315	1.2769	1	-0.02411	0.00304	0.78315	1.2769
Hemiplegia or paraplegia	1	0.00599	0.01914	0.92533	1.08069	1	0.09549	0.01611	0.92533	1.08069	1	-0.01084	0.01221	0.92533	1.08069	1	-0.0491	0.01107	0.92533	1.08069
Moderate/severe renal disease	1	0.01412	0.00752	0.50416	1.98349	1	0.05575	0.00632	0.50416	1.98349	1	0.01602	0.0048	0.50416	1.98349	1	-0.01412	0.00435	0.50416	1.98349
Liver disease	1	-0.02522	0.00575	0.82042	1.21889	1	-0.02658	0.00484	0.82042	1.21889	1	-0.00585	0.00367	0.82042	1.21889	1	-0.01765	0.00332	0.82042	1.21889
Diabetes	1	-0.00763	0.0054	0.46695	2.14156	1	0.00953	0.00454	0.46695	2.14156	1	0.00252	0.00344	0.46695	2.14156	1	-0.02165	0.00312	0.46695	2.14156
Cancer	1	0.07743	0.00875	0.43195	2.3151	1	0.03191	0.00736	0.43195	2.3151	1	0.03523	0.00558	0.43195	2.3151	1	0.01315	0.00506	0.43195	2.3151
Hypertension	1	0.01083	0.0041	0.55605	1.79841	1	0.0235	0.00345	0.55605	1.79841	1	0.00663	0.00262	0.55605	1.79841	1	-0.00296	0.00237	0.55605	1.79841
Hyperlipidemia	1	-0.03016	0.00429	0.62529	1.59927	1	-0.04074	0.00361	0.62529	1.59927	1	-0.00462	0.00274	0.62529	1.59927	1	-0.02593	0.00248	0.62529	1.59927
Hyperuricemia	1	0.01467	0.00897	0.93612	1.06824	1	0.00106	0.00755	0.93612	1.06824	1	-0.00299	0.00572	0.93612	1.06824	1	-0.00683	0.00519	0.93612	1.06824
Hyperthyroidism	1	-0.01674	0.01169	0.99197	1.0081	1	-0.01391	0.00984	0.99197	1.0081	1	-0.00512	0.00746	0.99197	1.0081	1	-0.01665	0.00676	0.99197	1.0081
Depressive disorder & anxiety	1	0.01337	0.00699	0.95895	1.04281	1	-0.01249	0.00588	0.95895	1.04281	1	0.00004957	0.00446	0.95895	1.04281	1	-0.01005	0.00404	0.95895	1.04281
Anemia	1	0.06521	0.00635	0.89914	1.11218	1	0.05932	0.00535	0.89914	1.11218	1	0.02508	0.00405	0.89914	1.11218	1	0.03761	0.00368	0.89914	1.11218
Parkinson's disease	1	0.10032	0.01213	0.91485	1.09307	1	0.10413	0.01021	0.91485	1.09307	1	0.00869	0.00774	0.91485	1.09307	1	0.04701	0.00702	0.91485	1.09307
Osteoporosis	1	-0.01197	0.0086	0.94697	1.056	1	0.00528	0.00724	0.94697	1.056	1	0.0018	0.00549	0.94697	1.056	1	0.00392	0.00498	0.94697	1.056
**Co-medications**																				
Nerve	1	0.03537	0.00464	0.84168	1.1881	1	0.03077	0.0039	0.84168	1.1881	1	0.00958	0.00296	0.84168	1.1881	1	0.0189	0.00268	0.84168	1.1881

Vascular	1	0.04802	0.00454	0.53203	1.87958	1	0.01825	0.00382	0.53203	1.87958	1	0.00785	0.0029	0.53203	1.87958	1	0.00956	0.00263	0.53203	1.87958
Metabolism	1	-0.01803	0.00464	0.49218	2.03176	1	-0.02808	0.0039	0.49218	2.03176	1	-0.00788	0.00296	0.49218	2.03176	1	-0.01466	0.00268	0.49218	2.03176
Others	1	0.01924	0.00291	0.79874	1.25197	1	-0.00564	0.00245	0.79874	1.25197	1	0.00823	0.00185	0.79874	1.25197	1	-0.00196	0.00168	0.79874	1.25197
